# Towards Achieving Sustainable Development: Role of Technology Innovation, Technology Adoption and CO_2_ Emission for BRICS

**DOI:** 10.3390/ijerph18010277

**Published:** 2021-01-01

**Authors:** Chi-Wei Su, Yannong Xie, Sadaf Shahab, Ch. Muhammad Nadeem Faisal, Muhammad Hafeez, Ghulam Muhammad Qamri

**Affiliations:** 1 School of Economics, Qingdao University, Qingdao 266071, China; 18561125566@163.com; 2 Department of Economics, Federal Urdu University of Arts Science and Technology, Islamabad 75300, Pakistan; shahab.sadaf@gmail.com; 3 Department of Computer Science, National Textile University, Faisalabad 37610, Pakistan; nadeem.faisal@ntu.edu.pk; 4 Faculty of Management & Administrative Sciences, University of Sialkot, Punjab 51080, Pakistan; muhammad.hafeez@uskt.edu.pk; 5 School of Economics, Nankai University, Tianjin 300071, China; gmqammar@hotmail.com

**Keywords:** technology innovation, technology adoption, BRICS, CO_2_ emission, Driscoll–Kraay panel regression

## Abstract

In the digital era, technology innovation and adoption trigger economic growth and enhance CO_2_ emissions through productivity, which places it in the mainstream policy debate. For BRICS economies, this paper uses the first method proposed in the literature to quantify their information and communication technology (ICT) and innovatively links each country to their information technology adoption rate, as a surrogate indicator for measuring information and communication technology. Environmental Kuznets curve evidence is also examined, using technology innovation, technology adoption, and trade openness as the control variables for sustainable development. The results show that two out of three technology innovation instruments, fixed telephone, and broadband subscriptions increase CO_2_ emissions. Simultaneously, mobile cellular subscriptions have a lowering effect on CO_2_ emission in BRICS. The technology adoption indicators, high-technology exports, and electric power consumption also cause an upsurge in CO_2_ emission. Moreover, trade openness also enriches the level of CO_2_ emission in the BRICS regions. There is a need to devise technology innovation and adoption policies to better use technology and to ensure a green environment.

## 1. Introduction

A growth structure with viability and sustainability is globally perceived as an operational target of all economies. To accomplish the target of high growth and sustainability is sometimes viewed as two distinct objectives, particularly when the role of technical progress is deemed as something that has depressing effects on the ecology [1]. As an aftermath, the struggle to achieve sustainable growth becomes an uphill task and challenges policymakers, thinkers, and researchers. The fast economic development of the past 30–35 years has been accompanied by natural resource depletion and declining environmental value [2]. The growth of the world economy at large has experienced a collapse of nature and social segregation. Consequently, the expansion of the green economy has steadily got the attention of researchers and authorities [3]. In the digital era, information, and communication technology (ICT) triggers energy efficiency and productivity. Innovation in the use of technology, particularly in the energy sector, is considered a solution to the environmental challenge of using technology sets [4]. The Global e-Sustainability Initiative (GeSI) believes that ICT will reduce 9 billion tons of carbon dioxide emissions by 2020 [5]. Likewise, Mathiesen et al. [6] and Ishida [7] suggested that ICT positively affects CO_2_ emissions. On the contrary, Salahuddin and Alam [8] argued that ICT enhances energy consumption and GHG. Similarly, the study of Van Heddeghem et al. [8] has mentioned that energy consumption from ICT products has increased from 3.9 to 4.6 percent in 2007–2012. Thus, the impact of ICT on CO_2_ emissions is still inclusive. Hence, the ICT and CO_2_ emissions nexus cannot be ignored.

The relationship between ICT and energy consumption and CO_2_ emissions is complex. In general, information technology has a positive impact on the quality of the environment, and energy and carbon dioxide emissions can be reduced through online delivery and transportation alternatives [5]. Asongu et al. [9] verified their impact on reducing environmental pollution by developing ICTs in 44 sub-Saharan African countries between 2002 and 2012. Gelenbe and Caseau [5] believed that ICT could continue to diminish energy consumption and CO_2_ emissions by changing jobs and business models. However, at the same time, many communication technology equipment and electronic waste will also harm the environment [10].

On the other side, the usage of electronic battery vehicles helps sustain the CO_2_ emissions in Europe [11]. The reduction of greenhouse gas and CO_2_ emissions also depends on energy sources and tax policy [12]. Furthermore, the possibility of subsidies and taxes also create the dynamics in CO_2_ emissions in Europe, considering each car segment and nation [12]; an increase can reduce the CO_2_ emissions through tariff subsidies for renewable energy [12]. In contrast, subsidies on CO_2_ utilization are helpful to uplift the oil production and carbon capture utilization and storage projects in China [13]. For the Chinese economy, taxes on coal production are also feasible to lower CO_2_ emissions [13].

Nocera et al. [13] suggested that the variation in social cost of CO_2_ emission for the transportation sector decreases by a single order of magnitude. Gelenbe and Caseau [5] examined the effect of ICT on the energy consumption and CO_2_ emission in different sectors. One the one hand, they found that ICT consumes energy and releases CO_2_ to affect the environmental atmosphere, but on the other hand, ICT can reduce CO_2_ emissions and the use of energy for other sectors, such as transportation, smart building, and virtual work and learning. Higón et al. [14] explored the association between ICT and CO_2_ emission. Unlike previous studies, this paper finds an inverted relationship between ICT and CO_2_ emission, and this result coincides with the opposite theory about the effect of ICT on CO_2_ emission. The finding, which is obtained by using a developing countries sample, can be applied to developed countries.

For Tunisia, Amri et al. [15] tested the environmental Kuznets curve (EKC henceforth) hypothesis and concluded that the EKC hypothesis does not apply to Tunisia. Moreover, his results explored an insignificant ICT influence on CO_2_ emission. Besides, trade and energy consumption have positive impacts on CO_2_ emission. Haseeb et al. [16] evaluated the effect of ICT, electricity consumption, economic growth, and financial development on environmental performance in BRICS countries. They found that these variables are connected and interacted together. For ICT, it can reduce CO_2_ emissions significantly and improve environmental quality. For the Japanese economy, Ishida [7] estimated that the long-run ICT investment elasticity of energy consumption is −0.155. He also argues that energy consumption can be declined moderately through ICT investment, but it will not increase the GDP. It was also shown that there is a nonlinear relationship between ICT and CO_2_ emissions, which follows an inverted U-shaped [17,18]. Only when information technology usage reaches the average level will it promote carbon dioxide emissions [14]. Therefore, the academic community has not yet reached a unified conclusion on the relationship between ICT and CO_2_ emissions. It is of great significance to rethink the impact of ICT on CO_2_ emissions using the latest methods and data.

How ICT achieves CO_2_ emission reduction targets has always been the focus of academic attention. The academic community generally believes that information and communication technologies are rapidly spreading through continuous innovation, affecting other economic sectors. Information and communication technologies are important drivers of new lifestyles and economic structural changes [19]. There are generally two ways to quantify this academic community: one is to quantify the internal communication technology of the enterprise, such as the technical efficiency, economic growth, industrial structure changes, etc. [20,21,22]; and the second is to establish a specific information technology impact model and establish an ICT environment level (ICT hardware life cycle), a second-level (ICT-provided services), and a third-level (emerging effect) impact model. It is believed that the dynamic impact of information technology comes from the feedback of the third-order effect on the first- and second-order effects [15,19].

In recent years, ICT technology in BRICS countries is also developing rapidly, greatly impacting its economic development [22,23]. Simultaneously, energy consumption and CO_2_ emissions are receiving increasing attention in Brazil, Russia, India, China, and South Africa (hereafter BRICS). Energy consumption also affects the economic policy uncertainty in the short term [23]. Because, according to data and forecasts from the International Energy Agency, the economy has beaten the OECD as the world’s largest emitter of carbon dioxide in 2013, and this growth trend will not be alleviated until 2023. Hence, it is valuable to study the relationship between ICT and CO_2_ emissions in BRICS countries. Nevertheless, the current academic research on this is still very limited; examples are exploring the impact of BRICS cooperation on the regional economy in the field of ICT [24], and the relationship between economic growth in BRICS countries and CO_2_ emissions [16]. However, there is no found relationship between ICT and environmental quality.

There is still only scant literature on CO_2_ emissions and innovation for a region or a group of BRICS countries. Innovation decreases the CO_2_ emission in some cross-sections of the countries but cannot help in others. For example, Dauda et al. [25] claimed that innovation played a role in lessening emissions in G6 countries, whereas it increased it in MENA and BRICS. For the Chinese industrial sector, Zhu et al. [26] estimated the effects of environmental regulations on technological innovation efficiency and recommended that the government publicize environmental regulations according to each province’s technological innovation capability. Generalizing this hypothesis for the BRICS economies can be the focus of an independent study. BRICS countries have shown an enormous growth episode in the past twenty years. On the other hand, they faced ecological issues, such as emitting GHGs, particularly CO_2_ emissions. The scorecard of BRICS economies in the Environmental Performance Index (EPI) 2018 suggests that most group members still have to do more to achieve the established policy goals on the environment. China (with 120), India (with 177), and South Africa (with 142) are still below the average score.

At present, the impact of ICT on the environment is mainly limited to global research. For the specific regions, such as the BRICS countries, the emergence of this paper fills the gap. The approach in this study is a holistic one because we have incorporated BRICS, which include almost half of the world’s population, and also because these countries have been involved in generating pollution more than any other group of countries. Three of them, China, India, and Russia, are found in the top four emission-generating countries [27]. The study of Köhler and Erdmann [17] recommended that scholars investigate the dynamic of new ICT applications and their depth effects on a global scale by reviewing the existing literature on ICT and GHG emission and putting forward future research about this topic after conducting an in-depth scenario analysis. This background suggests that a detailed empirical investigation is required to identify the role of adopting technology, technical value addition in manufacturing, and the resultant exports have in environmental degradation.

Hence, the paper studies the upshots of technology innovation and the adoption of CO_2_ emissions along the EKC for BRICS countries. The data suggest that all the BRICS countries are included in the top 15 most polluting countries. Accordingly, this paper uses the first method proposed in the literature to quantify ICT and innovatively links each country to their information technology adoption rate as a surrogate indicator for measuring the information and communication technology in BRICS economies. This study will also examine the environmental Kuznets’ curve evidence using technology innovation, technology adoption, and trade openness as the control variables.

## 2. Data Collection and Methodology

### 2.1. Model Specification

Based on existing literature, the present study establishes a linkage between technology innovation, technology adoption, and CO_2_ emission in BRICS economies. Salam et al. [28] argued that technological innovation and adoption trigger economic growth through human capital and skill, while the production level directly triggers CO_2_ emission [29]. Mobile phones and broadband are considered as core technologies of ICT [21]. Fixed broadband, fixed telephone, and mobile cellular subscriptions can be used to measure the ICT level of an economy [30]. Lee et al. [31] suggest that ICT also triggers human capital and skill, which increases economic growth. Likewise, ICT also enhances the investment level and international trade [32]. However, trade openness and value-added trade also upsurge CO_2_ emissions and air pollution indicators [33,34]. Similarly, Yao et al. [35] highlight that free trade agreements reduce bilateral CO_2_ emissions. Thus, the functional equation is as follows:

$$CO_{2}\;emission=f\left(Tech.\;Innovation,\;Tech.adoption,\;GDP,\;trade\;openness\right)$$

The natural logarithm form of the empirical model can be stated as

*Technology Innovation Model*

(1) $$LCE_{it}=\emptyset_{0}+\emptyset_{1t}Tech.Innovation_{it}+\emptyset_{2t}LG_{it}+\emptyset_{3t}LG2_{it}+\emptyset_{4t}TRO_{it}+\in_{1it}$$

*Technology Adoption Model*

(2) $$LCE_{it}=\emptyset_{5}+\emptyset_{6t}Tech.Adoption_{it}+\emptyset_{7t}LG_{it}+\emptyset_{8t}LG2_{it}+\emptyset_{9t}TRO_{it}+\in_{2it}$$

In Equations (1) and (2), technology innovation indicates the three instruments: fixed broadband (LFB), fixed telephone (LFT), and mobile cellular (LMC) subscriptions per 100 people, respectively, to gauge the technology innovation level of the economy [28,31,32]. Technology adoption is a measure of the high-technology exports (LHTE) and per capita electric power consumption (LEPC). Whereas *LCE* is the natural log of CO_2_ emission, *LG* is the natural log of GDP, *LG*2 is the square of the natural log of GDP, *TRO* is trade openness, “*i*” = BRICS economies, and “*t*” indicates the period. $\emptyset_{0},\;\mathrm{and}\;\emptyset_{5}$ indicate the intercepts of Equations (1) and (2), respectively, while $\emptyset_{1}\;\mathrm{to}\;\emptyset_{4}$ in Equation (1), and $\emptyset_{6}\;\mathrm{to}\;\emptyset_{9}$ in Equation (2), are the independent variable’s impact magnitudes.

This study is an effort to reveal the role of technology innovation and adoption in the CO_2_ emission of BRICS. To estimate the robust estimators, cross-sectional dependence is needed to be addressed in the computational method [36]. Therefore, the present study applies the DK (Driscoll–Kraay) estimation method to estimate the role of technology innovation and adoption on CO_2_ emission for BRICS economies. Hafeez et al. [37] and Özokcu et al. [38] recommend the DK regression method to tackle cross-sectional dependence issues of heteroskedasticity.

Besides this, the DK regression method has distinct advantages, such as providing robust estimators, dealing with missing values, and appropriate for both short and large time spans by tackling the spatial dependency and heteroskedasticity issues [39]. Even in the presence of spatial dependence and heteroskedasticity, the DK regression method computes the robust estimators [40]. Hence, the linear functional form of the DK method is

(3) $$LCE_{it}=\gamma_{0}+Z_{i,t}^{*}\rho \;+\varepsilon_{it}\;\;\;\;\;\;$$

Equation (3) indicates the “*i*” = BRICS countries, “*t*” is a period of the dataset, $LCE_{it}$ is a dependent variable (CO_2_ emission), $and\;Z_{i,t}^{*}$ is a set of control variables.

The CD tests reject the null hypothesis, concluding that the dataset of BRICS economies is cross-sectionally dependent. As a result, the panel unit root tests are not applicable [34]. Therefore, panel fixed effects regression and Wald test were applied to investigate the unit root in the BRICS panel [35]. For the panel fixed effects regression, the basic unit root mechanism is stated as follows:

(4) $$\pi_{it}=\emptyset \pi_{it-1}+\epsilon_{it}$$

Equation (4) shows that variable ($\pi_{it}$) is a function of its lag(s) ($\pi_{it-1}$). The null hypothesis, ∅=1, will be tested based on Wald statistics to diagnose the order of integration. The unit root results from the panel fixed effects regression and Wald statistic validate that there is no unit root process in the considered variables.

### 2.2. Sample Set and Description

To evaluate the role of technology innovation and adoption in CO_2_ emission (LCE), the study considers a panel dataset of BRICS economies covering the period 1990–2018. The selection of the period is based on the data availability of the considered variables. BRICS (Brazil, Russia, India, China, and South Africa) comprise a panel of major emerging economies of the world. The dataset was retrieved from WDI (World Development Indicators) [27]. Furthermore, CO_2_ emission (metric tons per capita) was used to quantify the environmental impact [41]. Hafeez et al. [30] suggest that CO_2_ emission has a higher ambient half-life than other air pollution indicators. Similarly, CO_2_ emission is also one of the major drivers of greenhouse gas through freight transport [35]. Gross domestic product (GDP) was taken from the GDP of 2010 in USD (LG) [42]. To gauge the technology innovation, three measurements, namely fixed broadband (LFB), fixed telephone (LFT), and mobile cellular (LMC) subscriptions per 100 people, were utilized, which have been suggested in the recent literature [32]. In turn, the high-technology exports as a percent of manufactured exports (LHTE) and electric power consumption at kWh per capita (LEPC) were used to measure the technology adoption [32]. The ratio of trade volume to GDP (as a percent of GDP) quantifies the trade openness (TRO) [42].

The natural log transformation was applied to the considered variables in this analysis to compute the elasticities of the estimates and for meaningful interpretation. Table 1 and Table 2 illustrate the descriptive statistics and correlation matrix of the BRICS economies, respectively. GDP, electric power consumption, and trade openness have higher mean values concerning the other considered variables of the study. Table 2 infers that electric power consumption, trade openness, and fixed telephone subscriptions have a more significant positive association with CO_2_ emission as compared to fixed broadband subscriptions, high-technology exports, and mobile cellular subscriptions. In comparison, the gross domestic product is negatively associated with CO_2_ emission.

## 3. Empirical Results and Discussions

### 3.1. Graphical Analysis

**Figure 1:**
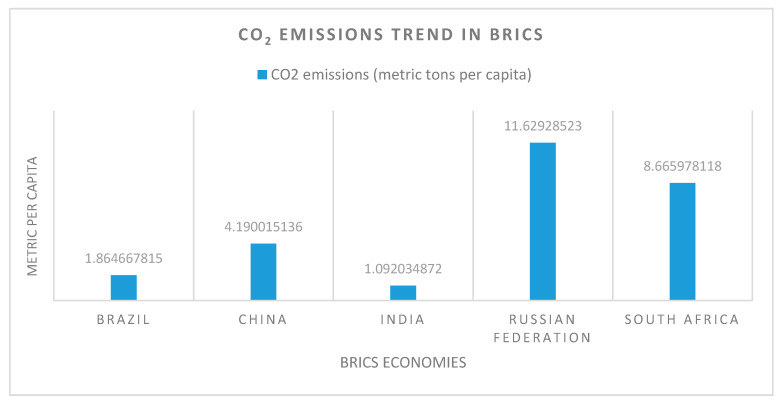
CO_2_ emission snapshot of BRICS [27].

**Figure 2:**
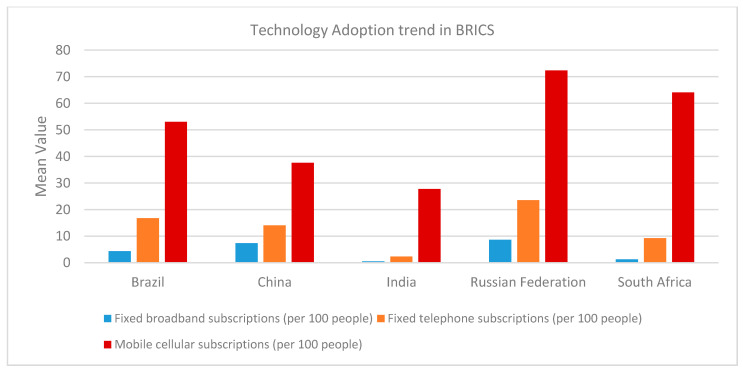
Technology innovation snapshot of BRICS [27].

The data suggest that all the BRICS countries are included in the top 15 most polluting countries. Three of them, China, India, and Russia, are found in the top four emission-generating countries [27]. Concerning an average per capita emission generation, Russia is on top of the BRICS group, followed by South Africa, China, Brazil, and India (Figure 1). China and India have less per capita emissions due to the large population as compared to other BRICS countries. Figure 2 depicts the recent technology adoption trend in BRICS economies.

**Figure 3:**
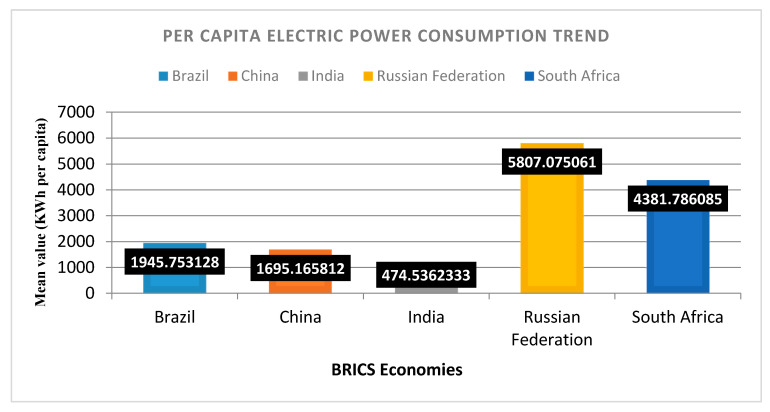
Technology adoption snapshot of BRICS: electric power consumption [27].

**Figure 4:**
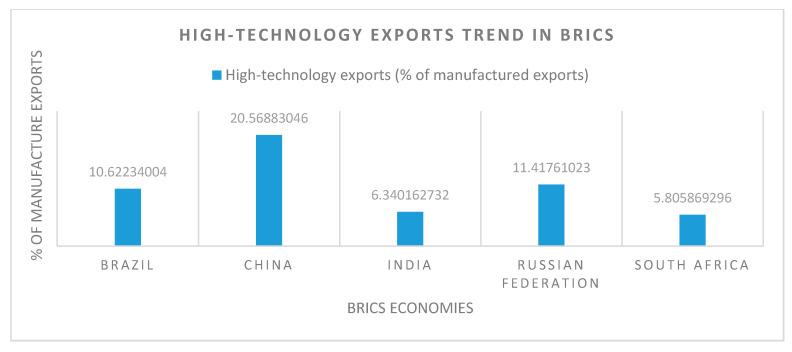
Technology adoption snapshot of BRICS: high-technology exports [27].

However, to the degree that energy use is relevant, Russia is again on top in consumption of per capita power, followed by South Africa, China, Brazil, and India, due to being highly populated countries. So, the use of energy and emitting CO_2_ are correlated, and we can hypothesize that energy use increases the risk of environmental degradation [13]. ICT is mitigating greenhouse gas (GHG) emissions and recovers environmental quality [6]. Russia also has an edge in the adoption of technology over China, Brazil, South Africa, and India (Figure 3). As far as technology use in the value addition for manufactured exports is concerned, China is on top of the BRICS group, followed by Russia, Brazil, India, and South Africa (Figure 4).

### 3.2. Preliminary Testing for Panel Analysis

In the panel dataset, the cross-sectional dependence (CD) issue needed to be examined as a preliminary test before the investigation of the panel unit tests [43,44]. The results of the CD tests are reported in Table 3. The CD tests reveal the cross-sectional dependence degree among the input variables—fixed telephone, broadband, and mobile cellular subscriptions; GDP, trade openness, high-technology exports, electric power consumption, and the output variables of CO_2_ emissions in the BRICS panel. The CD tests reject the null hypothesis, concluding that the dataset of the BRICS economies is cross-sectionally dependent. As a result, the panel unit root tests are not applicable [35]. Therefore, panel fixed effects regression and Wald tests were applied to investigate the unit root in the BRICS panel in Table 4.

### 3.3. Empirical Analysis and Results Discussion

The technology innovation estimators from DK and the Newey–West standard error method reveal interesting outcomes and are illustrated in Table 5. For robustness checking and estimating strength, the Newey–West standard error method is used and depicted at the bottom of Table 5. The estimates unfold that 2 out of 3 instruments of technology innovation, LFT and LFB, have a significantly positive impact on the CO_2_ emissions of BRICS. It signifies that fixed broadband and telephone subscriptions have a dominant impact on CO_2_ emission. The estimates infer that a 1% increase in fixed broadband and fixed telephone subscriptions will drive a 0.502% and 0.056% increase in the per capita CO_2_ emissions in the BRICS group, respectively. In the digital era, technology innovation, as a key pillar of the digital economy, creates employment opportunities, improves the living standard, and triggers business development [32]. In contrast, business development and production levels are contributing a significant addition to CO_2_ emission [29,30].

On the contrary, a 1% increase in mobile cellular subscriptions will decrease the per capita CO_2_ emissions by 0.1835%, but it is statistically insignificant. It infers that mobile cellular subscriptions have a lowering effect on CO_2_ emissions in BRICS economies, which is supported by the results of [9]. ICT is considerably reducing the CO_2_ emissions in sub-Sharan African countries, as learnt from interactive regressions [9]. The BRICS economies are focusing on energy-efficient ICT to speed up the internet and mitigate the energy consumption to sustain environmental quality. Similarly, Chavanne et al. [45] also argue that ICT behaves as a constructive instrument of environmental quality through GHG mitigation. Matsumoto et al. [46] have elaborated that the more advanced the changing ubiquitous networking technologies are, the more the CO_2_ emissions decrease.

Furthermore, trade openness has a significantly positive impact on the CO_2_ emission of the BRICS region. The study of Rauf et al. [47] suggests that trade openness creates a rise in energy consumption, resulting in environmental degradation. Furthermore, the EKC hypothesis is tested, and the U-Shape EKC hypothesis is validated in technology innovation models, which is in line with the studies of [30]. The U-Shape EKC hypothesis indicates that GDP reduces the CO_2_ emission at the initial stages, and CO_2_ emission starts to increase in later stages of development, which is a line with the study of Yasmeen et al. [34]. The Newey–West regression validates the estimates from the DK regression and is reported at the bottom of Table 5.

Table 6 depicts the outcome of the technology adoption model from DK and the Newey–West standard error method. Energy usage creates an environmental degradation risk [48]. The electricity consumption stimulates CO_2_ emissions [49]. Therefore, the total energy consumption upsurges the CO_2_ emission and carbon footprints [37]. Likewise, the energy disparities are also upsurging the CO_2_ emission in the One Belt and Road region (BRI) [50]. Likewise, technology innovation models and technology adoption models also provide evidence of the U-Shape EKC hypothesis in the BRICS region, in line with the work of [19,20]. The magnitude of high-technology exports and electric power consumption is 0.118 and 0.701, respectively, implying that both technology adoption measures positively impact the CO_2_ emission of the BRICS region [26]. The estimates also reveal that the impact of electric power consumption is higher than high-technology exports on CO_2_ emission.

GDP has a statistically negative impact on CO_2_ emission in the BRICS region. The significant positive magnitude of the square of GDP (LG2) indicates that CO_2_ emissions will start to increase at a later stage of development [34]. The EKC hypothesis was tested, and the U-Shape EKC hypothesis was validated in the technology adoption models, which is in line with the studies of [30]. The U-Shape EKC hypothesis indicates that GDP reduces the CO_2_ emission at initial stages; the CO_2_ emissions start to increase in later stages of development, which is a line with the study of [34]. Hafeez et al. [50] briefly examined the energy use, trade openness, and higher income levels in China, concluding that environmental deterioration has been critical in China due to fast growth in the last few decades. Lastly, the coefficient of trade openness is 0.661, implying that a 1% increase in trade openness will enrich the BRICS CO_2_ emission by 0.66%. Value-added trade upsurges the air pollution indicators [40]. The study of Hafeez et al. [50] has studied the energy–growth nexus and found that energy consumption, economic growth, and trade openness reduce pressure on energy demand after the threshold income level for the BRICS countries. On the contrary, Yao et al. [36] point out that free trade agreements are a better strategy to reduce bilateral CO_2_ emissions. The Newey–West regression validated the estimates from the DK regression and are reported at the bottom of Table 6.

## 4. Conclusions

The prime objective of this paper is to unfold the first method proposed in the literature to quantify ICT, to innovatively link each BRICS economy to their information technology adoption rate as a surrogate indicator for measuring information and communication technology, using the most recent available dataset from 1990 to 2018. This study also examined the environmental Kuznets’ curve evidence using technology innovation, technology adoption, and trade openness as the control variables. The CD tests were applied to identify the degree of cross-sectional dependence among the BRICS countries. To tackle the cross-sectional dependence, the unit root test by panel fixed effects regression was applied and found that the study variables have no unit root process. The DK standard error method was applied to compute robust parameters for both the technology innovation and adoption models in the presence of cross-sectional dependence. The result estimates of both the technology innovation and adoption models were validated through the Newey–West standard error method. The empirical estimates shed light on insightful outcomes and validate the U-shape EKC hypothesis in technology innovation and adoption models for the BRICS region. In technology innovation models, fixed broadband and fixed telephone subscriptions have a significant positive impact on CO_2_ emission. In comparison, mobile cellular subscriptions have a lowering effect on CO_2_ emissions in BRICS economies. The BRICS economies are focusing on energy-efficient ICT to speed up the internet and mitigate their energy consumption to sustain environmental quality. Technology adoption indicators, high-technology exports, and electric power consumption have a statistically significant positive effect on CO_2_ emissions for BRICS economies. Trade openness also enhances the CO_2_ emissions in BRICS, both for the technology innovation and adoption models. Furthermore, GDP has a significant role in the technology–CO_2_ emission nexus.

This study signifies that technology innovation and adoption are suitable instruments to sustain the environment through ICT reforms. The results from technology innovation and adoption may devise possible policy suggestions, such as (i) policymakers encouraging mobile cellular subscriptions to decrease the CO_2_ emissions; (ii) to tackle the positive influence of energy consumption on CO_2_ emissions, allocate the implementation of renewable energy resources, and energy conservation projects for a better quality of the environment; (iii) the introduction of eco-friendly technology can also decline risks of environmental degradation; and (iv) the U-shape EKC hypothesis suggests that policymakers may consider the later stages of development.

The results of the BRICS region can be generalized for other case studies by applying the same theory. Additional specifications are needed, such as the cross-sectional dependence, spatial dependency, and heteroskedasticity issues of the considered region. Future research may consider the energy matrix and CO_2_ emission of each technology because each country’s energy matrix is important and should be considered to compare the CO_2_ emission by each technology.

## Figures and Tables

**Table 1:** Descriptive statistics.

Variable	Obs	Mean	Std. Dev.	Min	Max
LCE	145	1.325	0.933	−0.34	2.638
LEPC	145	7.597	0.956	5.61	8.806
LFB	145	−0.125	2.534	−7.439	3.29
LFT	145	2.207	1.027	−0.539	3.46
LG	145	27.804	0.909	26.099	29.95
LHTE	145	2.233	0.546	1.372	3.429
LMC	145	1.792	3.48	−8.504	5.112
TRO	145	3.648	0.416	2.719	4.706

Note: Obs, Mean, Std. Dev., Min, and Max indicate the total number of observations, average value, standard deviation, minimum value, and maximum value, respectively. Source: Compiled from [27].

**Table 2:** Matrix of correlations.

Variables	(1)	(2)	(3)	(4)	(5)	(6)	(7)	(8)
(1) LCE	1.000							
(2) LEPC	0.921	1.000						
(3) LFB	0.379	0.395	1.000					
(4) LFT	0.636	0.8	0.482	1.000				
(5) LG	−0.181	−0.14	0.391	0.297	1.000			
(6) LHTE	0.198	0.189	0.395	0.572	0.752	1.000		
(7) LMC	0.204	0.29	0.559	0.459	0.315	0.281	1.000	
(8) TRO	0.715	0.528	0.364	0.331	−0.095	0.198	0.395	1.000

Note: (1) LCE, (2) LEPC, (3) LFB, (4) LFT, (5) LG, (6) LHTE, (7) LMC, and (8) TRO. Source: Calculated from dataset 1990–2018 of [27].

**Table 3:** Cross-sectional dependence analysis.

Cross-Sectional Dependence (CD) Test				Cross-Sectional Dependence (Based on Residuals)	
Null hypothesis: cross-section are independent				Null hypothesis: errors of the cross-section are independent	
**Variable**	**CD-stats**	**Variable**	**CD-stats**		
LCE	7.326 *	LG	15.423 *	Pesaran’s test	−2.596 *
	(0.00)		(0.00)		(0.0094)
LEPC	9.548 *	LG2	15.444 *	Friedman’s test	12.756 **
	(0.00)		(0.00)		(0.0125)
LFB	11.258 *	LHTE	2.927 *	Frees’ test	0.450 *
	(0.00)		(0.003)	Critical values from Frees’ Q distribution	
LFT	9.621 *	LMC	16.072 *	alpha = 0.10	0.0892
	(0.00)		(0.00)	alpha = 0.05	0.1160
TRO	8.159 *			alpha = 0.01	0.1660
	(0.00)				

**Note:** () indicates the probability values; * and ** shows the 1 and 5% level of significance, respectively.

**Table 4:** Unit root analysis.

	LCE	LFT	LMC	LFB	LHTE	LEPC	LG	LG2	TRO
LCE (−1)	0.513 ^a^								
LFT (−1)		0.474 ^a^							
LMC (−1)			0.531 ^a^						
LFB (−1)				0.437 ^a^					
LHTE (−1)					0.527 ^a^				
LEPC (−1)						0.546 ^a^			
LG(−1)							0.808 ^a^		
LG2(−1)								0.795 ^a^	
TRO(−1)									0.359 ^a^
Wald Test									
Chi^2^	123.58 ^a^	135.17 ^a^	116.65 ^a^	185.88 ^a^	126.62 ^a^	91.68 ^a^	12.53 ^a^	13.92 ^a^	166.99 ^a^

Note: “a” indicates the level of significance at 1%.

**Table 5:** Technology innovation model.

	Technology Innovation Model			
Variables	LFT Model	LMC Model	LFB Model	All
LFT	0.502 * (0.00)	-	-	0.534 * (0.00)
LMC	-	−0.1835 (0.519)	-	−0.079 * (0.00)
LFB	-	-	0.084 *** (0.09)	0.056 ** (0.04)
LG	−6.442 * (0.004)	−7.587 * (0.00)	−6.636 * (0.00)	−6.860 * (0.00)
LG2	0.110 * (0.006)	0.134 * (0.00)	0.1152 * (0.00)	0.118 * (0.00)
TRO	1.057 * (0.00)	1.555 * (0.00)	1.293 * (0.00)	1.166 * (0.00)
Constant	90.178 * (0.00)	102.465 * (0.00)	91.930 * (0.00)	95.283 * (0.00)
R^2^	0.7838	0.5492	0.5818	0.8379
RMSE	0.4401	0.6354	0.6120	0.3838
F-Stats	149.57 (0.00)	154.87 (0.00)	200.95 (0.00)	72.67 (0.00)
BRICS	5	5	5	5
Observation	145	145	145	145
**Newey–West standard error method**				
LFT	0.502 * (0.00)	-	-	0.534 * (0.00)
LMC	-	−0.0183 (0.375)	-	−0.079 * (0.00)
LFB	-	-	0.084 * (0.003)	0.056 * (0.001)
LG	−6.44 * (0.00)	−7.587 * (0.00)	−6.636 * (0.001)	−6.86 * (0.00)
LG2	0.110 * (0.00)	0.134 * (0.00)	0.115 * (0.001)	0.118 * (0.00)
TRO	1.057 * (0.00)	1.555 * (0.00)	1.293 * (0.00)	1.166 * (0.00)
Constant	90.17 * (0.00)	102.46 * (0.00)	91.93 * (0.001)	95.28 * (0.00)
F-Stats	192.59 (0.00)	117.89 (0.00)	92.61(0.00)	146.46 (0.00)
BRICS	5	5	5	5
Observation	145	145	145	145

**Note:** () indicates the probability values; *,**, and *** shows the 1, 5, and 10% level of significance, respectively.

**Table 6:** Technology adoption model.

	Technology Adoption Model		
Variables	LHTE Model	LEPC Model	All
LHTE	0.619 * (0.001)	-	0.118 *** (0.1)
LEPC	-	0.721 * (0.00)	0.701 * (0.00)
LG	−5.43 * (0.002)	−3.044 * (0.002)	−2.756 ** (0.011)
LG2	0.09 * (0.005)	0.053 * (0.003)	0.047 ** (0.016)
TRO	1.297 * (0.00)	0.683 * (0.00)	0.661 * (0.00)
Constant	76.486 * (0.002)	36.354 * (0.007)	33.06 ** (0.02)
R^2^	0.5912	0.9271	0.9287
RMSE	0.6051	0.2555	0.2537
F-Stats	151.86 (0.00)	1020.35 (0.00)	447.20 (0.00)
BRICS	5	5	5
Observation	145	145	145
**Newey–West standard error method**			
LHTE	0.619 * (0.00)	-	0.118** (0.04)
LEPC	-	0.72 * (0.00)	0.701 * (0.00)
LG	−5.43 * (0.002)	-	−2.756 * (0.001)
LG2	0.09 * (0.004)	−3.044 * (0.00)	0.047 * (0.002)
TRO	1.297 * (0.00)	0.683 * (0.00)	0.661 * (0.00)
Constant	76.48 * (0.003)	36.35 * (0.00)	33.06 * (0.00)
F-Stats	105.38 (0.00)	538.88 (0.00)	504.71(0.00)
BRICS	5	5	5
Observation	145	145	145

**Note:** () indicates the probability values; *,**, and *** shows the 1, 5, and 10% level of significance, respectively.

## Data Availability

The data presented in this study are available on request from the corresponding author.

## Abbreviations

CD = cross-sectional dependence; CO_2_ = carbon emissions; EKC = environmental Kuznets curve; DK = Driscoll–Kraay standard error method; EPI = environmental performance index; GDP = gross domestic product; GHG = greenhouse gas; ICT= information and communication technology; LCE = log of CO_2_ emissions; LG = log of GDP; LG2 = log of square of GDP; LFB = log of fixed broadband subscriptions; LFT = log of fixed telephone subscriptions; LMC = log of mobile cellular subscriptions; LHTE = the high-technology exports; LEPC = log of per capita electric power consumption; TRO = trade openness; WDI = world development indicator; BRICS = Brazil, Russia, India, China, and South Africa.
